# *Brain Communications* 2023 early career researcher paper prize

**DOI:** 10.1093/braincomms/fcad335

**Published:** 2023-12-29

**Authors:** Tara L Spires-Jones

**Affiliations:** Edinburgh, UK

## Abstract

Our editor invites nominations for the early career researcher paper prize for an article published in *Brain Communications* in 2023.

## Graphical Abstract

**Graphical Abstract:**
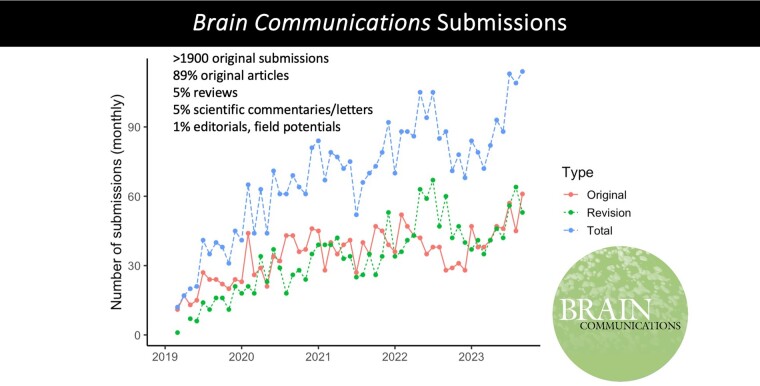

Welcome to Volume 6, issue 1 of *Brain Communications*. In this Editorial, I’m proud to announce the call for nominations for the second annual *Brain Communications* early career researcher paper prize. As in our inaugural prize last year,^1^ the aim is to recognize the contributions of an early career or technical researcher for their research. Our first winner was Dr George Thomas for his paper ‘Changes in both top-down and bottom-up effective connectivity drive visual hallucinations in Parkinson’s disease’.^2^ George delivered an excellent talk at the Brain Conference about this work^3^ in addition to receiving a gift voucher and an invitation to the annual Guarantors of Brain dinner. Now, we are seeking nominations for the first author of a paper published in 2023 in *Brain Communications* to win this fabulous prize. If you know of a deserving researcher (student, postdoc or technician) who published as a first author with us in the last year, please fill in this short nomination form by the end of January 2024. Please note before nominating that the winner will be invited to give a talk at the Brain Conference in person on 15 March 2024 in London (https://guarantorsofbrain.org/news/brain-conference-2024/), so please confirm with the nominee that they would be able to present their work (in person or potentially online), before submitting the nomination. Our editorial board members will vote and the winner will be announced in February 2024.

In another initiative to support early career researchers, the Guarantors of Brain have agreed to support a stipend for a summer internship for a student to work with *Brain Communications* in the summer of 2024. Keep an eye on our social media for invitations to apply for this opportunity.

Of course, in addition to our early career researcher initiatives, we continue to publish excellent translational neuroscience papers. We recently accepted a study by Chen and colleagues describing a recessive mutation in *SCN1B* in children with epilepsy, which encodes a voltage-gated sodium channel β1. Authors introduced the mutant *SCN1B-*c.265C>T gene into mice resulting in epilepsy and sudden death in the animals, generating an important model for the field.^4^ Due to the strong papers submitted by our authors, our reputation continues to grow and our submissions are increasing (see *Graphical Abstract*). Many thanks to all of our authors for sending us their excellent work.

The cover image for this issue comes from Accogli *et al*.^5^ and shows an illustration of the ‘ears of the lynx’ neuroimaging sign present in several neurological disorders including people with a neurodevelopmental disorder caused by *LNPK1* mutations causing lunapark deficiency, described in their paper (cover image courtesy of Valentina Turchetti).
